# Characteristics of Japanese Older Adults Whose Trunk Muscle Mass Decreased during the COVID-19 Pandemic

**DOI:** 10.3390/ijerph191811438

**Published:** 2022-09-11

**Authors:** Tamaki Hirose, Yohei Sawaya, Masahiro Ishizaka, Naori Hashimoto, Akihiro Ito, Yoshiaki Endo, Kaoru Kobayashi, Akihiro Yakabi, Ko Onoda, Tsuyoshi Hara, Miyoko Watanabe, Masafumi Itokazu, Akira Kubo, Tomohiko Urano

**Affiliations:** 1 Department of Physical Therapy, School of Health Sciences, International University of Health and Welfare, 2600-1 Kitakanemaru, Otawara 324-8501, Tochigi, Japan; 2 Senior Services Division of Otawara, 1-4-1 Honcho, Otawara 324-8641, Tochigi, Japan; 3 Department of Geriatric Medicine, School of Medicine, International University of Health and Welfare, 4-3 Kozunomori, Narita 286-8686, Chiba, Japan

**Keywords:** body composition, COVID-19, frailty, sarcopenia, SARS-CoV-2 infection

## Abstract

The coronavirus (COVID-19) pandemic significantly affected the physical and mental functions in older adults, resulting in “corona-frailty”. This 2-year prospective study characterized changes in quantitative measures and corona-frailty among a cohort of community-dwelling older women. Changes were evaluated using face-to-face interactions with 39 Japanese women (mean age: 76.1 ± 5.9) in 2019 (pre-pandemic baseline) and 2021 (follow-up during the pandemic). Quantitative measurements of handgrip strength, walking speed, calf circumference, body composition, and background factors were evaluated. Body weight and trunk muscle mass significantly decreased at follow-up. Multiple regression analysis, using change in trunk muscle mass as the dependent variable and background factors as independent variables, identified that decrease in trunk muscle mass was associated with “being robust at baseline” and answering “Yes” to the question of “Do you go out less frequently compared with last year”? The 2-year trunk muscle mass change for each baseline frailty stage showed a significant decrease only in the robust group (−8.0%). The decrease in trunk muscle mass might be related to pandemic-induced lifestyle restraint, suggesting that robust older adults who are healthy and active should take measures that focus on trunk muscles to avoid “corona-frailty”.

## 1. Introduction

The coronavirus disease 2019 (COVID-19), which has spread worldwide since 2020, has forced lifestyle changes upon people and limited their activities [1]. The mortality and serious illness rates due to COVID-19 infection were reported to be high in older adults who were forced to engage in self-restrained living [2,3,4]. These restrictions led to the avoidance of group gatherings, previously recommended to reduce isolation and anxiety and to promote exercise and communication among older adults. Specifically, community activities were reduced. The negative effect of COVID-19 control measures on the physical and mental functioning of older adults has been labeled “corona-frailty” [5].

Studies have shown that aging in older adults is associated with a decline in grip strength, walking speed, muscle strength, and muscle mass [6,7,8,9,10]. Hence, we hypothesized that these characteristics of aging would become more severe due to “corona-frailty” during the COVID-19 pandemic which would affect people who were classified originally as having frailty; that is, those who were more vulnerable. However, previous studies on the COVID-19 pandemic used questionnaire-based surveys to minimize face-to-face interactions [5,11,12,13]. Few reports used quantitative measurements, such as handgrip strength [14], and fewer comparisons were made with pre-COVID-19 data.

To contribute toward the development of countermeasures to frailty during the pandemic, especially on the physical approach to be taken, this study used face-to-face measurements taken before and during the COVID-19 pandemic to compare the characteristics of corona-frailty among community-dwelling older women.

## 2. Materials and Methods

### 2.1. Study Design and Participants

This was a 2-year prospective cohort study conducted in Otawara, Tochigi, Japan from 2019 (baseline, before the COVID-19 pandemic) to 2021 (follow-up, during the pandemic). The interval between baseline and follow-up was 733.7 ± 89.0 days (mean days ± standard deviation). Recruitment of participants was performed by written notifications from Otawara City Hall to the representatives of “Kayoi-no-ba” in each community, wherein the representatives notified the community residents [15]. In both the baseline and follow-up surveys, the participants were those who voluntarily participated in the physical check-up programs sponsored by the city.

**Figure 1:**
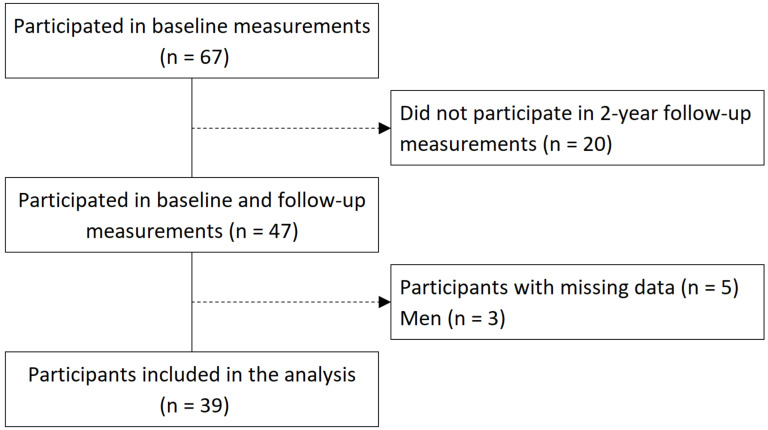
Flowchart of participant recruitment.

Following the inclusion criteria, 67 participants were initially included in the baseline measurements. Among them, 20 participants who did not follow-up, as well as three men and five women with missing data were all excluded from the study (Figure 1). Thus, only 39 community-dwelling women (mean age: 76.1 ± 5.9 years) were included in the analysis. None of the participants in this study had difficulty understanding instructions and expressing their intentions.

### 2.2. Body Composition, Handgrip Strength, Walking Speed, and Calf Circumference

All assessments were performed by 12 qualified physiotherapists with at least 6 years of experience. Body composition was measured using the multifrequency bioelectrical impedance analysis method (MC-780A-N, TANITA, Tokyo, Japan) [16]. Body weight, body mass index (BMI), body fat, body fat percentage, appendicular skeletal muscle mass (ASM), arm muscle mass, leg muscle mass, and trunk muscle mass were also measured. ASM index (ASMI) was calculated by dividing ASM by the square of the height. Handgrip strength was measured twice in the standing position using a Smedley-type hand dynamometer (TKK 5401, Takei Scientific Instruments; Niigata, Japan). The maximum value was considered the representative value. Usual walking speed was measured on a 6 m walking path with a 1 m acceleration and deceleration path. Calf circumference was measured once on each side in the sitting position, and the maximum value was considered representative. Previous studies have shown high intra- and inter-rater reliabilities for handgrip strength, walking speed, and calf circumference [17,18,19,20,21].

### 2.3. Evaluation of Frailty

Frailty was assessed at baseline and follow-up using the Kihon Checklist (KCL), a questionnaire consisting of 25 items in 7 areas: activities of daily living, physical function, nutrition, oral function, outdoor activity, cognitive function, and depression. The KCL has been reported to be a reliable and valid assessment index for frailty, and its use in diagnosing frailty is strongly recommended in the Asian Frailty Guidelines [22,23,24,25,26]. The KCL was judged with a score of 0–3 indicating robust, 4–7 indicating pre-frailty, and ≥8 indicating frailty [22,27].

### 2.4. Evaluation of Background Factors

Background factors included whether the participant lived alone, the number of prescribed medications taken, the presence/absence of sports or physical exercise, and opportunities to go out [28,29]. The respondents were asked to mark “Yes” or “No” to each of the following questions: “Do you engage in low levels of physical exercise aimed at health”?, “Do you engage in moderate levels of physical exercise or sports aimed at health”?, and “Do you go out less frequently compared with last year”? In addition, the progression of frailty was noted over two years. Progression was defined as a deterioration of at least one level of frailty stage. Participants who were diagnosed with sarcopenia according to the Asian Working Group for Sarcopenia 2019 criteria were noted [30].

### 2.5. Statistical Analysis

Body composition, handgrip strength, walking speed, and calf circumference were compared using a paired *t*-test at baseline and follow-up. ASMI was analyzed using the Wilcoxon signed-rank test. Multiple regression analysis was performed using the change in trunk muscle mass, which showed a significant difference between baseline and follow-up, as the dependent variable and the evaluation of background as the independent variable. The independent variables, which are listed in background factors, were entered using a stepwise method. Changes in trunk muscle mass by frailty stage were compared between baseline and follow-up using a paired *t*-test. All paired *t*-tests were performed after confirming a normal distribution of measurement values using the Kolmogorov–Smirnov test/Shapiro–Wilk test. All significance levels were set at 5%. IBM SPSS Statistics version 25.0 (IBM Japan; Tokyo, Japan) was used for all statistical analyses. The study power was analyzed using G*Power version 3.1.9.2 [31].

## 3. Results

Among the participants, at the baseline, 53.8% had robust (*n* = 21), 30.8% pre-frailty (*n* = 12), and 15.4% frailty (*n* = 6) statuses. Table 1 shows the changes in body weight and trunk muscle mass during the follow-up period as analyzed using the paired *t*-test.

Table 2 shows the background factors analyzed in this study. The mean number of medications taken was 2.9 ± 2.5 at baseline and 3.4 ± 2.6 at follow-up. The number of medications in baseline frailty status was 2.0 ± 2.2 for robust participants, 3.8 ± 2.1 for pre-frailty, and 4.3 ± 3.1 for frailty. Nine participants were living alone. Three participants at baseline and five at follow-up marked no for the question on low levels of physical exercise, and ten participants at baseline and twelve at follow-up marked no for the question on moderate levels of physical exercise or sports. Sixteen participants marked yes for the question on less frequent outings compared with last year. One patient had sarcopenia at baseline, and nine patients deteriorated in the frailty stage over 2 years.

**Figure 2:**
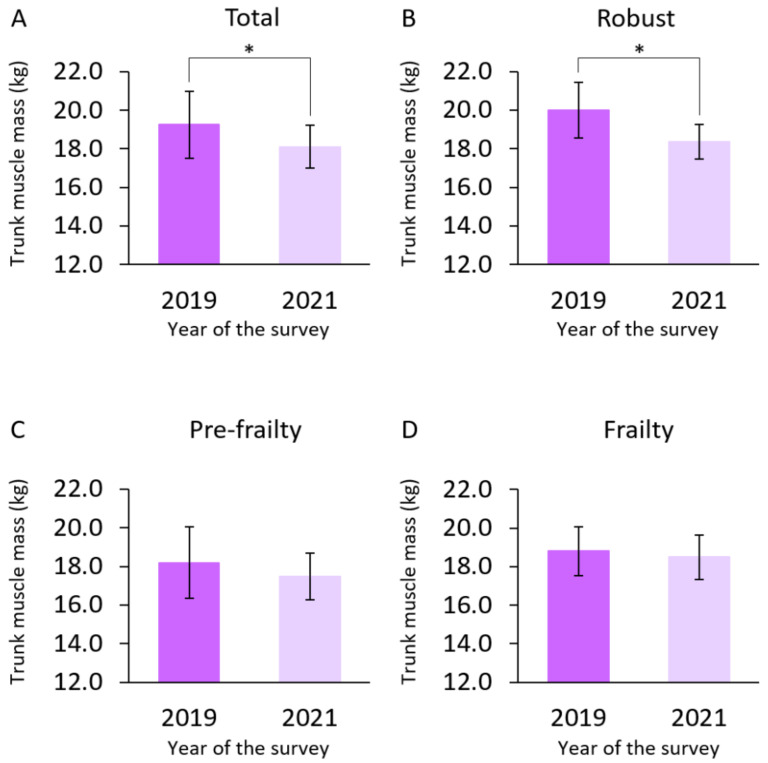
Change in trunk muscle mass by frailty status between baseline and follow-up. The bars represent mean ± standard deviation for the (**A**) total, (**B**) robust, (**C**) pre-frailty, and (**D**) frailty groups. Classified by baseline frailty status (in 2019), trunk muscle masses in 2019 and 2021 were compared using paired *t*-tests. A significant decrease was observed in the robust group but not in the pre-frailty or frailty groups. In the study period, 2019 corresponds to before the onset of the COVID-19 pandemic, and 2021 corresponds to during the pandemic; * *p* value < 0.05.

Table 3 shows multiple regression analyses with the change in trunk muscle mass as the dependent variable and the background factors as the independent variables. Having a robust baseline status and “going out less frequently compared to the previous year” were identified as factors contributing to trunk muscle mass reduction. The residuals of the regression model showed a normal distribution in the Kolmogorov–Smirnov and Shapiro–Wilk tests. Multiple regression analyses showed an appropriate sample size, given a post hoc power of 0.82 via F tests calculated from an effect size of 0.28 [32]. Figure 2 shows the results of the paired *t*-test for trunk muscle mass for each baseline frailty group. The mean trunk muscle mass was 20.0 ± 1.4 and 18.4 ± 0.9 kg (robust), 18.2 ± 1.8 and 17.5 ± 1.2 kg (pre-frailty), and 18.8 ± 1.3 and 18.5 ± 1.2 kg (frailty), in 2019 and 2021, respectively, with a significant decrease in the robust (*p* < 0.001) group. Conversely, the pre-frailty (*p* = 0.067) and frailty (*p* = 0.455) groups showed no significant decrease.

Multiple regression analyses used the change in trunk muscle mass as the dependent variable and the background factors as the independent variables. Two independent variables were selected using a stepwise method. The following independent variables were not selected: age, living alone, the number of medications, presence/absence of sarcopenia, “Do you engage in moderate levels of physical exercise or sports aimed at health”? at baseline and follow-up, “Do you engage in low levels of physical exercise aimed at health”? at baseline and follow-up, and deterioration of frailty status.

## 4. Discussion

This 2-year longitudinal study reports a decrease in trunk muscle mass in community-dwelling older Japanese women during the study period, which coincided with a period of life restraint due to the COVID-19 pandemic which was consistent with a study by Son et al. that recently reported a significant decrease in the trunk muscle mass of women during COVID-19 [14]. Based on this, it can be inferred that a decrease in trunk muscle mass is a characteristic change during the COVID-19 pandemic. In addition to this, the novelty of our study was the association of the decreased trunk muscle mass with a “robust stage”, at baseline and “going out less frequently compared to last year”.

The reduction in trunk muscle mass over two years was 8.0% in the robust group, which was higher than the reported values of about a 1–2% decrease per year and about 10% over 40 years after age 40 [33,34]. Generally, the lower extremities show the greatest loss of muscle mass with aging [34,35,36]. Considering that the decrease in muscle mass reported in this study was higher than the change from aging alone and involved different areas of muscle mass loss than that observed in older adults, it can be inferred that the combination of age-related changes and the self-restraint in living due to the COVID-19 pandemic may have further reduced the amount of daily activity relevant to trunk area compared to other areas. The 30% decrease in physical activity and a corresponding increase in sedentary lifestyle, owing to social distancing and isolation during the pandemic, caused corona-frailty [37]. In addition, the change in trunk muscle mass differed from our hypothesis that originally frail people would be more susceptible to corona-frailty and instead demonstrated the change in the more robust group. It has been shown that the risk of developing new frailty was greater during the COVID-19 pandemic [38,39] and a decrease in muscle mass (or body weight) has been associated with faster frailty progression [40]. Our study suggests that corona-frailty countermeasures should focus on active robust individuals to prevent the development of new frailty.

Reduced opportunities to go outside in conventional life are reported to affect long-term functional changes in older adults, and low muscle mass is associated with impairing social life including going outside [41,42,43]. Declining trunk muscle strength is caused by long-term inactivity [44]. It has already been reported that the older adults during the COVID-19 pandemic period decreased their frequency of going out, especially women, and that they were aware of their physical and mental decline due to voluntarily refraining from this activity [45,46]. Even during the COVID-19 pandemic, maintaining social participation is a key strategy for preventing sarcopenia and frailty [14]. In our study, the decrease in trunk muscles was observed in those who were less likely to go out at the follow-up compared to the previous year which suggests that the period of self-restraint due to the COVID-19 pandemic may have intensified the decrease.

This study had both advantages and limitations. First, there were differences in skeletal muscle mass based on sex. Because of the small initial number of male participants, only females were included in the analysis. However, the study remains valid because their baseline muscle mass (arms, legs, and trunk) was similar to that found for participants in the same age groups in previous studies [35]. Moreover, both the number of medications by total participants and each frailty status were similar to those in previous studies, showing general numbers that are comparable to those in the present study [7,47,48,49]. Second, the sample size was small; considering COVID-19 control measures, it was difficult to conduct a large-scale study. Third, it is possible that the results of this study are not attributable to the COVID-19 pandemic, since they could not be compared with a non-pandemic situation. Fourth, since this physical check-up program was managed by the Otawara City Hall, it was difficult to obtain the medical history and control the baseline and follow-up periods. Despite these limitations, the major advantage of this study is that the evaluation was performed using face-to-face interactions despite the study period overlapping the COVID-19 pandemic in Japan. It is unclear when the COVID-19 pandemic will end in Japan, and further changes in physical function and worsening in frailty status are expected to occur in its aftermath, requiring continued observation.

## 5. Conclusions

Our findings suggest that the key to preventing physical function decline in older adults during COVID-19 may be to approach the trunk in more active individuals, as well as ensure that they have opportunities to go outside, while taking care to prevent infection.

## Figures and Tables

**Table 1:** Comparison of measurements between baseline and follow-up.

	Baseline	Follow-Up	*p* Value
Body weight (kg)	53.1 ± 8.3	52.0 ± 8.5	0.010 *
BMI (kg/m^2^)	23.6 ± 3.7	23.4 ± 3.9	0.285
Body fat (kg)	17.8 ± 6.6	17.7 ± 6.8	0.859
Arm fat (kg)	1.5 ± 0.7	1.5 ± 0.7	0.798
Leg fat (kg)	6.5 ± 2.1	6.7 ± 2.1	0.168
Trunk fat (kg)	9.8 ± 3.9	9.6 ± 4.2	0.259
Body fat percentage (%)	32.5 ± 7.9	33.0 ± 8.3	0.413
Calf circumference (cm)	33.5 ± 2.8	33.5 ± 2.8	0.889
Handgrip strength (kg)	23.2 ± 3.7	23.0 ± 3.4	0.413
Usual walking speed (m/s)	1.3 ± 0.2	1.4 ± 0.3	0.382
ASMI (kg/m^2^) †	6.3 ± 0.7	6.4 ± 0.5	0.118
ASM (kg)	14.1 ± 1.8	14.3 ± 1.8	0.468
Arm muscle mass (kg)	3.2 ± 0.4	3.1 ± 0.4	0.516
Leg muscle mass (kg)	11.0 ± 1.5	11.2 ± 1.5	0.324
Trunk muscle mass (kg)	19.3 ± 1.7	18.1 ± 1.1	<0.001 *

* *p* value < 0.05, values were reported as mean ± standard deviation; †: ASMI was analyzed using the Wilcoxon signed-rank test; the other variables were analyzed using the paired *t*-test; BMI: body mass index; ASMI: appendicular skeletal muscle mass index; ASM: appendicular skeletal muscle mass.

**Table 2:** Assessment of background factors at baseline and follow-up.

Baseline			
Age	76.1 ± 5.9		
Number of medications	2.9 ± 2.5		
“Do you engage in low levels of physical exercise aimed at health?”	Yes (36)	No (3)	
“Do you engage in moderate levels of physical exercise or sports aimed at health?”	Yes (29)	No (10)	
Sarcopenia	No sarcopenia (38)	Sarcopenia (1)	
Frailty status	Robust (21)	Pre-frailty (12)	Frailty (6)
**Follow-Up**			
Number of medications	3.4 ± 2.6		
Living alone	Yes (9)	No (30)	
“Do you go out less frequently compared with last year?”	Yes (16)	No (23)	
“Do you engage in low levels of physical exercise aimed at health?”	Yes (34)	No (5)	
“Do you engage in moderate levels of physical exercise or sports aimed at health?”	Yes (27)	No (12)	
Deterioration of frailty stage	Presence (9)	Absence (30)	

Values were reported as mean ± standard deviation. (Number of participants).

**Table 3:** Multiple regression analysis of the change in trunk muscle mass.

	β	95% CI	*p* Value
Frailty status at baseline †	0.714	0.101 to 1.327	0.024 *
“Do you go out less frequently compared with last year”? at follow-up ‡	−0.933	−1.852 to −0.014	0.047 *

* *p* < 0.05; CI: confidence interval; †: robust = 0; pre-frailty = 1; frailty = 2; ‡: No = 0; Yes = 1.

## Data Availability

Research data are not shared.
